# Trajectory Tracking Control of High-Speed Vehicles on Wet and Slippery Roads

**DOI:** 10.3390/s25175450

**Published:** 2025-09-03

**Authors:** Xiaohua Song, Kuifeng Chen, Yujia Zheng, Xiaoyan Zhang

**Affiliations:** School of Electronic Information Engineering, Xi’an Technological University, Xi’an 710021, China; chenkuifengxatu@st.xatu.edu.cn (K.C.); zhengyujia@st.xatu.edu.cn (Y.Z.); xyzhang@xatu.edu.cn (X.Z.)

**Keywords:** trajectory tracking control, MPC (Model Predictive Control), PID (Proportional-Integral-Derivative) control, high-speed vehicles, wet and slippery roads

## Abstract

**Highlights:**

**What are the main findings?**

- In response to the limited existing research on high-speed autonomous driving under low-adhesion, slippery road conditions, a novel MPC-PID fused trajectory tracking control algorithm is proposed. This approach significantly enhances tracking accuracy and vehicle stability under such complex scenarios.
- Joint simulation results demonstrate that the proposed method improves trajectory tracking accuracy by at least 22.2%, while comfort, fuel economy, and safety are improved by 9.4%, 19.8%, and 5.3%, respectively.

**What is the implication of the main finding?**

- This work provides theoretical support and simulation validation for high-speed trajectory tracking control under low-adhesion conditions, offering high engineering applicability and practical significance.
- The proposed approach facilitates real-time, precise, and robust autonomous driving in dynamically complex environments, promoting the deployment of high-performance control strategies in real-world applications.

**Abstract:**

Autonomous vehicle trajectory tracking control is one of the hot topics in the autonomous driving field. One of the most widely used control methods is MPC (Model Predictive Control). As the control system generally becomes more nonlinear and complex, more nonlinear system factors are added to the MPC method. However, tracking accuracy and the amount of calculation needed are both dependent on a lot of contradictions for NMPC (Nonlinear Model Predictive Control). This research proposes a control algorithm for MPC-fused PID (Proportional-Integral-Derivative) control that ensures tracking accuracy under different high-speed driving conditions on wet and slippery road surfaces. The objective of the algorithm is twofold: first, to enhance trajectory tracking accuracy, and second, to ensure real-time control and optimize the vehicle’s comfort, economy, and safety indexes. The results of the joint simulation in Carsim/MATLAB Simulink show that trajectory tracking accuracy is improved by at least 22.2% under high-speed driving conditions of a vehicle on a wet and slippery road. At the same time, the comfort, economy, and safety of the vehicle are improved by at least 9.4%, 19.8%, and 5.3%, respectively.

## 1. Introduction

Trajectory tracking control is one of the most important research topics in autonomous driving. To ensure safe and reliable operation, autonomous vehicles must be capable of maintaining accurate trajectory tracking under a variety of operating conditions. However, most existing studies focus on conventional and relatively simple driving scenarios. In practice, high-speed driving on wet and slippery roads is a common yet challenging condition. Under such circumstances, road adhesion significantly decreases as vehicle speed increases, making vehicles more susceptible to phenomena such as hydroplaning. Therefore, it is essential to investigate trajectory tracking control strategies that address the complexities of high-speed driving under low-adhesion conditions.

Stability control in trajectory tracking is one of the core challenges in the field of autonomous driving, and currently the main way to improve the driving stability of vehicles in complex environments is through optimizing the control algorithm [1,2,3] and constructing a high feasibility reference trajectory [4,5,6]. The former typically employs advanced methodologies, such as model predictive control or nonlinear robust control, with the objective of mitigating lateral sway and trajectory tracking errors during vehicle operation. In contrast, the latter emphasizes the generation of smooth and dynamically feasible reference trajectories, taking into account the vehicle’s dynamic constraints and environmental sensing.

The most widely used control methods are PID (Proportional-Integral-Derivative) control, MPC (Model Predictive Control), and LQRs (Linear Quadratic Regulators) [7,8,9,10,11,12,13]. PID control is a control method with a negative feedback controller based on the compensation deviation. It does not require a precise control model. The control effect is achieved by adjusting the controller parameters [14,15]. However, the determination of PID controller parameters requires a lot of experimental conditions; the control effect changes greatly after the experimental conditions are changed. In modern control theory, the control object of LQRs is a linear system given in the form of state space; the objective function is a quadratic function of object state and control input. The optimal control of LQRs means that the designed state feedback controller should make the quadratic objective function take the minimum [16,17]. The MPC method is characterized by the predictive model, the rolling optimization, and feedback correction. It is suitable for situations where many constraints need to be conducted and an accurate model cannot be established [18]. In the actual driving process, it is necessary to predict future driving behavior through current environments with complicated road and traffic conditions; but the PID control method and LQRs do not have this advantage. But the MPC method can realize the predictions needed for the real-time controls and calculations. Thus, LTV-MPC (Linear Time-Varying Model Predictive Control) was proposed and applied due to its simple linearization and fast calculation speed [19]. Many studies have established mechanical models for different research objects. They have obtained good trajectory tracking control effects by using the LMPC (Linear Model Predictive Control) method [19,20,21,22,23]. Gallep et al. [24] designed an MPC for lateral vehicle guidance, which calculated an optimal manipulated variable sequence for an inner low-level steering angle controller. The MPC controller can ensure accurate and robust path tracking during different maneuvers and speeds. However, when the control object gradually becomes a complex nonlinear system, the accuracy of the MPC controller becomes worse. Thus, more nonlinear factors should be added to the MPC controller [25]. Zietkiewicz et al. [26] compared the two control methods of NMPC (Nonlinear Model Predictive Control) and FBL (Feedback Linearization) + MPC. The two methods are equivalent in terms of control accuracy, but the latter has a faster calculation speed. Therefore, the choice between these two methods depends on the achieved accuracy of the solution and the afforded calculation time in each step of predictive control. All current research regarding the NMPC algorithm are focused on balancing control accuracy and calculation time [27,28,29,30,31,32,33].

The primary goal of path tracking control for autonomous vehicles is to achieve high-precision trajectory tracking under complex conditions while ensuring real-time performance. This requires balancing control accuracy with computational efficiency. Common strategies include the optimal model, prediction horizon adjustment, control cycle tuning, and hybrid control structures. Although the optimal model strategy reduces computational time by lowering model complexity or applying linearization, it often sacrifices accuracy under dynamic conditions. Adjusting the predictive time domain (*N*) can optimize performance for specific scenarios but may require real-time adaptation in diverse environments. Similarly, control period tuning can improve efficiency, though overly low frequencies risk delayed responses—especially in MPC, where responsiveness is critical for safety. In contrast, increasing PID frequency improves accuracy up to a point, beyond which the computational cost rises sharply. Hybrid control structures combine different algorithms to balance precision and efficiency. Though they may not achieve optimal performance in both aspects, they do ensure acceptable accuracy within a reasonable computational time. A comparative overview of these methods is summarized in Table 1.

Trajectory tracking in autonomous vehicles relies on environmental data gathered by onboard sensors and the vehicle’s planned path. By precisely controlling the propulsion and steering systems, the vehicle follows the designated trajectory while ensuring safety. Trajectory tracking serves as a core indicator of an autonomous vehicle’s overall control performance.

This study focuses on the challenge of maintaining accurate trajectory tracking at high speeds on low-adhesion road surfaces. Under such conditions, reduced tire–road friction can lead to side slip, significant lateral deviation, and even complete loss of control during trajectory execution.

In order to improve the tracking reliability of autonomous vehicles at high-speed conditions on wet and slippery roads, a more accurate vehicle dynamics model is used as a predictive model; and a model predictive control algorithm is used to design the tracking controller to improve the controller’s ability to predict the future behavior of the vehicle. The accuracy of trajectory tracking is ensured. Trajectory tracking accuracy is maximized, as are the three indicators of comfort, economy, and safety under high-speed driving conditions of vehicles on wet and slippery road surfaces. This is achieved by means of appropriate vehicle dynamics modeling and joint control methods.

The operation of autonomous vehicles at high speeds, especially on wet and slippery surfaces, imposes strict constraints on actuator control inputs to ensure stability and optimal performance. Skidding is primarily caused by the loss of tire–road friction, while rollover is driven by excessive lateral acceleration. Both phenomena are known to compromise vehicle stability, particularly under high-speed conditions. To address these challenges, it is essential to incorporate rigorous nonlinear dynamic constraints into the control framework. While MPC offers robust predictive capabilities, it alone may fail to meet trajectory tracking accuracy requirements under certain extreme conditions. Therefore, combining MPC with PID control can enhance overall system performance and improve tracking robustness across a wider range of scenarios.

Our contributions in this work are summarized as follows:

- Path tracking study of autonomous vehicles under high-speed driving conditions on wet and slippery roads. At present, there is a lack of in-depth research on these kinds of working conditions in the field of autonomous vehicles, but solving any issues related to these conditions is crucial for the promotion of autonomous vehicles in the future, and this study effectively fills this research gap, which is of great significance and value to engineering practice;
- Joint controller design of MPC and PID control. By effectively combining the advantages of MPC’s high accuracy and PID control’s low response delay, the joint controller shows excellent performance under high-speed driving conditions on slippery roads. Moreover, due to the real-time adjustable parameters of PID control, the joint controller has good scalability and has strong potential for other complex tracking paths and complex working conditions;
- Comprehensive evaluation index for multi-objective co-optimization. In this study, the comprehensive evaluation indexes of path tracking accuracy, comfort, economy, and safety are constructed, which can provide an objective and comprehensive performance evaluation of the designed controllers as well as of multiple sets of controller parameters, vehicle speeds, and roadway adhesion coefficients.

## 2. Control Strategies for High-Speed Driving on Wet and Slippery Roads

The subject of path tracking control of autonomous vehicles traveling at high speeds on wet and slippery roads belongs to a kind of path tracking control research under complex working conditions. In such working conditions, autonomous vehicles are required to make accurate control decisions in a brief time frame while also ensuring the stability of the vehicle. This could pose a significant challenge to the real-time and robustness of the control system of the autonomous vehicle. The high accuracy and predictable characteristics of the MPC control algorithm show considerable potential for future application in autonomous vehicle control. However, the computational demands of this approach can be substantial, which may limit its applicability in real-time scenarios, where rapid response is crucial. The PID control algorithm boasts a number of merits, including its straightforward architecture, its rapid response, and its capacity for real-time parameter adjustments. These attributes have led to its extensive utilization in contemporary control systems. Consequently, the integration of MPC and PID control emerges as a pivotal strategy within the context of this study’s application scenario. This practical success is of significant value and importance in the field of autonomous vehicles due to its strong engineering practice implications.

**Figure 1:**
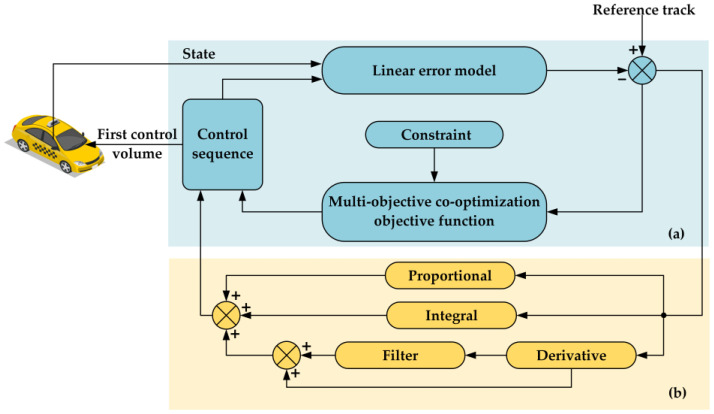
Joint controller structure of MPC and PID control. (**a**) is the internal structure of MPC; (**b**) is the internal structure of PID.

In order to achieve highly stable and real-time trajectory tracking while meeting the requirements of accuracy, safety, comfort, and economy, a joint controller of MPC and PID control has been developed. The configuration of the controller is illustrated in Figure 1.

**Figure 2:**
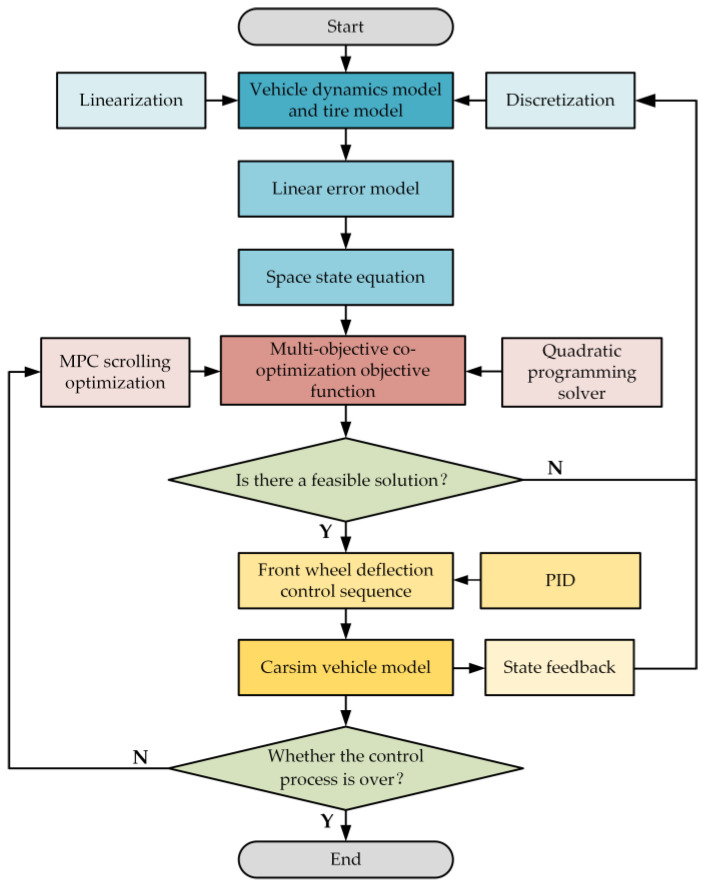
Overall process framework of control system.

In the context of a joint controller of MPC and PID control, the vehicle dynamics model initially receives feedback regarding the vehicle’s state quantities. Subsequent to the linearization and discretization of this model, a linear error model is extracted, which is then immediately converted into a spatial state equation. Subsequently, the multi-objective co-optimization objective function in the MPC controller is solved using a quadratic programming solver. The result is then evaluated. In the event that the solution is non-feasible, the vehicle dynamics model is returned to for the subsequent round of solving. Conversely, if the solution is feasible, the control sequence of the vehicle’s front wheel deflection angle is the output. At this juncture, the PID controller receives the difference from the linear error model and the reference trajectory and compensates the control sequence output by the MPC controller to form the final control sequence output by the joint controller. Thereafter, the vehicle executes the first control quantity in the control sequence. The termination of the vehicle’s path tracking control process is determined through a series of assessments. In the event that the process has not yet reached its conclusion, the multi-objective co-optimization objective function solution in the MPC controller is revisited in order to establish the rolling optimization of the joint controller. Conversely, if the process has been fully completed, the entire control process is considered to have reached its conclusion. The control flowchart of the joint MPC and PID controller is depicted in Figure 2.

## 3. Joint Controller Design for MPC and PID Control

The objective of this study is to explore the design concept of a joint controller that integrates the merits of both the MPC and the PID controllers. The primary goal is to leverage the precision offered by MPC and the real-time capability of PID control to enhance the performance of path tracking control in autonomous vehicles operating at high speeds on wet and slippery surfaces, which represent a challenging operational scenario due to their complexity. The MPC controller is capable of solving the multi-objective co-optimization objective function under constraints and generating the control sequence for the front wheel angle of the autonomous vehicle. The PID controller, on the other hand, is responsible for outputting the control volume for the front wheel angle of the autonomous vehicle, which is based on the difference between the linear error between the model and the reference trajectory. Furthermore, the PID controller outputs the front wheel turning angle control quantity for the autonomous vehicle and compensates the output result of the MPC controller. This joint approach effectively addresses the issue of the MPC controller’s inability to respond promptly to changes in the state quantity of autonomous vehicles, which can lead to traffic accidents due to the prolonged calculation time.

### 3.1. MPC Controller Design

**Figure 3:**
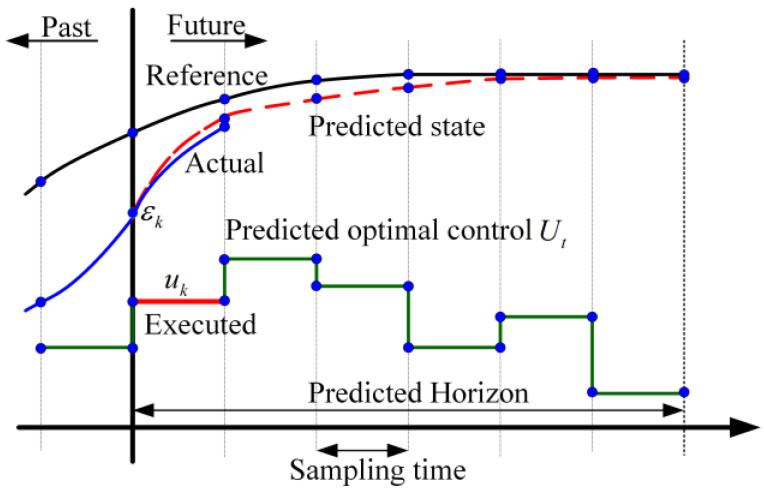
Schematic of MPC.

MPC offers strong control performance and robustness. It is particularly effective in dealing with system uncertainties, nonlinearities, and multivariable interactions. Furthermore, MPC is well suited for systems where numerous constraints must be simultaneously satisfied in both control inputs and system outputs. A diagram of the MPC controller’s principal design is shown in Figure 3.

The MPC model comprises three fundamental components: predictive modeling, rolling optimization, and feedback correction. A salient benefit of the MPC approach is its capacity to manage constraints in an implicit manner. This objective is accomplished by employing the model to forecast the system’s future dynamic behavior and subsequently integrating the constraints directly into the optimization problem for future inputs, outputs, or states. These constraints are part of a secondary programming problem that can be solved online in real time. The control structure is composed of three primary components: a linear error model, a multi-objective co-optimization objective function, and constraints. The linear error model is extracted from the vehicle dynamics model and is discretized and linearized to obtain the spatial state equations. The optimization problem is then solved using a quadratic programming-based solver. The structure of the MPC controller in the combined MPC and PID controller is shown in Figure 1a.

#### 3.1.1. Linear Error Model

The objective of trajectory tracking control in autonomous vehicles is to ensure accurate tracking under high-speed conditions on wet and slippery road surfaces. To meet this objective, the vehicle dynamics model must balance accuracy and computational efficiency. Therefore, an appropriate level of model simplification is applied while retaining the essential dynamics relevant to trajectory tracking performance.

The following assumptions are made in the modeling process:

- The vehicle travels on a flat road surface with no vertical motion;
- The suspension and vehicle body are considered rigid;
- The coupling between longitudinal and lateral tire forces is neglected, and only lateral slip behavior is modeled;
- A 3-DOF (three-Degree-Of-Freedom) bicycle model is used to describe lateral, longitudinal, and yaw motion;
- Load transfer between the front and rear axles is ignored;
- Vehicle speed is assumed to vary slowly, and aerodynamic effects in both directions are neglected.

**Figure 4:**
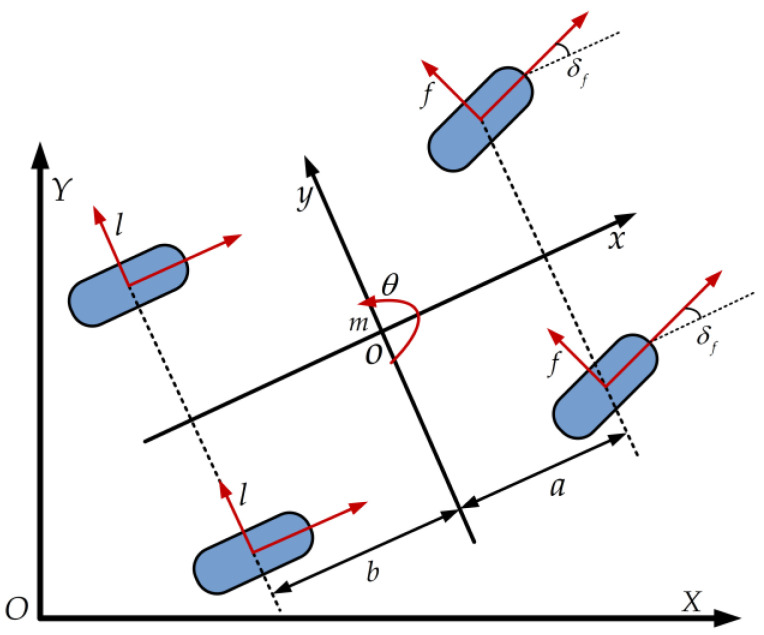
Schematic diagram of vehicle dynamics model.

Based on these assumptions, the vehicle dynamics model is constructed as illustrated in Figure 4.

The nonlinear vehicle dynamics model based on the assumption of a small front wheel deflection angle and a linear tire model [34,35] is shown in Equation (1).

(1) $$\left\{\begin{array}{l} m\ddot{y}=-m\dot{x}\dot{\theta }+2\left[f+l\right]\\ m\ddot{x}=m\dot{y}\dot{\theta }+2\left[C_{lf}s_{f}+f\delta_{f}+C_{lr}s_{r}\right]\\ I_{Z}\ddot{\theta }=2\left[af-bl\right]\\ \dot{Y}=\dot{x}\sin\theta +\dot{y}\cos\theta \\ \dot{X}=\dot{x}\cos\theta -\dot{y}\sin\theta \\ f=C_{cf}\left[\delta_{f}-\left(\dot{y}+a\dot{\theta }\right)/\dot{x}\right]\\ l=C_{cr}\left(b\dot{\theta }-\dot{y}\right)/\dot{x} \end{array}\right.$$

where m is the mass of the vehicle; a and b are respectively the length from the center of mass to the front or rear axles; f is the lateral force received by the front wheels; l is the lateral force received by the rear wheels; x and y are respectively the lateral and longitudinal displacements of the vehicle; $I_{z}$ is the moment of inertia of the vehicle around the Z axis; θ is the yaw angle of the vehicle body; $C_{cf}$ and $C_{cr}$ are respectively the lateral stiffness of the front wheels and the rear wheels; $\delta_{f}$ is the front wheel slip angle; $s_{f}$ is the front wheel slip rate; $s_{r}$ is the rear wheel slip rate; $C_{lf}$ is the longitudinal stiffness of the front wheel; $C_{lr}$ is the longitudinal stiffness of the rear wheel; and X and Y are the coordinates of the front and rear axles of the vehicle, respectively.

The relationship between the state and control quantities for any reference system is shown in Equation (2).

(2) $${\dot{\hat{\xi }}}_{r}=f({\hat{\xi }}_{d},u_{r})$$

The nonlinear vehicle dynamics model must be linearized to facilitate controller design. By performing a Taylor series expansion around an operating point and neglecting higher-order terms, an LTV (Linear Time-Varying) model can be obtained. The resulting expression for the linearized system is shown in Equation (3).

(3) $${\dot{\hat{\xi }}}_{d}=A_{d}\left(t\right){\hat{\xi }}_{d}\left(t\right)+B_{d}\left(t\right)u_{d}\left(t\right)$$

In this model, state quantity is set to $\xi_{d}={\left[\dot{y},\dot{x},\theta ,\dot{\theta },Y,X\right]}^{T}$. The control value is selected as $u_{d}=\delta_{f}$. The output value is selected as $\eta_{d}={\left[\theta ,Y\right]}^{T}$. During high-speed trajectory tracking, only the front wheel steering angle is controlled, with the vehicle maintaining a constant longitudinal speed. Autonomous vehicles impose stringent real-time performance demands on the controller under such high-speed conditions.

Equation (3) is discretized to obtain the discrete space state expression:

(4) $$\xi_{d}(k+1)=A_{d}(k)\xi_{d}(k)+B_{d}(k)u_{d}(k)$$

(5) $$A_{d}(k)=I+TA_{d}(t),\;B_{d}(k)=TB_{d}(t)$$

The expression of each component in Equation (4) is shown in Equation (5), and $B_{d}\left(t\right)$ and $A_{d}\left(t\right)$ are shown as Equations (6) and (7).

(6) $$B_{d}\left(t\right)=\left[\frac{2C_{cf}}{m},\frac{C_{cf}\left(\delta_{f,t-1}-\frac{\dot{y}+a\dot{\varphi }}{\dot{x}}\right)}{m},0,\frac{2aC_{cf}}{I_{z}},0,0\right]$$

(7) $$A_{d}\left(t\right)=\left[\begin{array}{cccccc} \frac{-2(C_{cf}+C_{cr})}{m{\dot{x}}_{t}} & \frac{\partial f_{\dot{y}}}{\partial \dot{x}} & 0 & -{\dot{x}}_{t}+\frac{2(bC_{cf}-aC_{cr})}{m{\dot{x}}_{t}} & 0 & 0\\ \dot{\varphi }-\frac{C_{cf}\delta_{f,t-1}}{m{\dot{x}}_{t}} & \frac{\partial f_{\dot{x}}}{\partial \dot{x}} & 0 & {\dot{y}}_{t}-\frac{2aC_{cf}\delta_{f,t-1}}{m{\dot{x}}_{t}} & 0 & 0\\ 0 & 0 & 0 & 1 & 0 & 0\\ \frac{2(bC_{cr}-aC_{cf})}{I_{z}{\dot{x}}_{t}} & \frac{\partial f_{\dot{\varphi }}}{\partial \dot{x}} & 0 & \frac{-2(b^{2}C_{cr}+a^{2}C_{cf})}{I_{z}{\dot{x}}_{t}} & 0 & 0\\ \cos\varphi_{t} & \sin\varphi_{t} & {\dot{x}}_{t}\cos\varphi_{t}-{\dot{y}}_{t}\sin\varphi_{t} & 0 & 0 & 0\\ -\sin\varphi_{t} & \cos\varphi_{t} & -{\dot{y}}_{t}\cos\varphi_{t}-{\dot{x}}_{t}\sin\varphi_{t} & 0 & 0 & 0 \end{array}\right]$$

In the formula, the specific expressions of $\frac{\partial f_{\dot{\varphi }}}{\partial \dot{x}}$, $\frac{\partial f_{\dot{y}}}{\partial \dot{x}}$, and $\frac{\partial f_{\dot{x}}}{\partial \dot{x}}$ are shown in Equation (8).

(8) $$\left\{\begin{array}{l} \frac{\partial f_{\dot{\varphi }}}{\partial \dot{x}}=(2aC_{cf}({\dot{y}}_{t}+a{\dot{\varphi }}_{t})-2bC_{cr}\left({\dot{y}}_{t}-b{\dot{\varphi }}_{t}\right))/I_{z}{\dot{x}}_{t}^{2}\\ \frac{\partial f_{\dot{y}}}{\partial \dot{x}}=(2aC_{cf}({\dot{y}}_{t}+a{\dot{\varphi }}_{t})+2C_{cr}\left({\dot{y}}_{t}-b{\dot{\varphi }}_{t}\right))/m{\dot{x}}_{t}^{2}-{\dot{\varphi }}_{t}\\ \frac{\partial f_{\dot{x}}}{\partial \dot{x}}=(2C_{cf}\delta_{f,t-1}({\dot{y}}_{t}+a{\dot{\varphi }}_{t}))/(m{\dot{x}}_{t}^{2}) \end{array}\right.$$

#### 3.1.2. Multi-Objective Co-Optimization Objective Function and Constraints

Since the selected vehicle model is dynamic, it inherently possesses greater complexity than a kinematic model and involves more constraints. As a result, during controller execution, it is possible that the optimal solution cannot be found within the specified time when multiple constraints are simultaneously active. To address this issue, a relaxation factor is introduced to the objective function. The primary purpose of this modification is to ensure the tracking performance of the controller. The optimized objective function is shown in Equation (9).

(9) $$J(\xi_{d}(t),u_{d}\left(t-1\right),\Delta U_{d}\left(t\right))={\sum_{t=1}^{N_{P}}||\eta_{d}(t+i\left|t\right.)-\eta_{d,ref}(t+i\left|t\right.)||}_{Q}^{2}+{\sum_{t=1}^{N_{C-1}}\left\|\Delta u_{d}(t+i\left|t\right.)\right\|}_{R}^{2}+\rho \varepsilon^{2}$$

In the formula, $\eta_{d}$ is the output, $\eta_{d,rdf}$ is the desired output, and the first part of objective function is mainly to satisfy tracking accuracy. The second part is mainly to meet the stability and comfort of tracking and prevent sudden changes in the control variables. The third part of objective function is the relaxation factor.

To prevent the occurrence of no optimal solution within the specified time, the feasible set is appropriately relaxed. The constraints imposed to optimize the objective function are presented in Equation (10).

(10) $$\begin{array}{l} s.t.\\ \;\left\{\begin{array}{l} \Delta U_{d,\min}\leq \Delta U_{d,t}\leq \Delta U_{d,\max}\\ U_{d,\min}\leq A\Delta U_{d,t}\leq U_{d,\max}\\ y_{hardc,\min}\leq y_{hardc}\leq y_{hardc,\max}\\ y_{softc,\min}-\varepsilon \leq y_{softc}\leq y_{softc,\max}+\varepsilon \\ \varepsilon >0 \end{array}\right. \end{array}$$

In the formula, $y_{hardc}$ is the output for hard constraints. Thus, for the output quantity whose constraint range cannot be changed during the control process, $y_{hardc,\min}$ and $y_{hardc,\max}$ are the hard constraint output limit values. Furthermore, $y_{softc}$ is the soft constraint output. Thus, for the output quantity whose constraint range can be changed during the control process, $y_{softc,\min}$ and $y_{softc,\max}$ are the soft constraint output limit values.

In each control cycle, the optimization objective function is solved, and the sequence of control increments and relaxation factors in the control time domain are obtained, as shown in Equation (11).

(11) $$\Delta {U^{*}}_{d,t}={\left[\Delta u_{d,t}^{*},\Delta u_{d,t+1}^{*},\ldots ,\Delta u_{d,t+N_{c}-1}^{*},\varepsilon \right]}^{T}$$

The first control quantity of the control sequence for the front wheel angle of the autonomous vehicle is shown in Equation (12).

(12) $$u_{d}(t)=u_{d}(t-1)+\Delta u_{d,t}^{*}$$

This process is repeated in each subsequent control cycle until the trajectory tracking control task is completed.

During the design of the model predictive controller, in order to ensure that the tire operates within its linear region, the tire cornering angle must be restricted. Additionally, dynamic constraints related to the vehicle model—such as the vehicle’s center of mass slip angle, stability conditions, and tire slip angle constraints—must be incorporated. The specific constraints are detailed as follows.

(1) Lateral acceleration of vehicle.

The power of a vehicle is influenced not only by its driving force but also by the tire–road adhesion conditions. Therefore, vehicle–road attachment constraints are incorporated into the high-speed trajectory tracking model. The longitudinal and transverse accelerations are limited by ground adhesion and can be shown in Equation (13).

(13) $${\left(a_{x}^{2}+a_{y}^{2}\right)}^{0.5}\leq \mu g$$

In the formula, $a_{x}$ and $a_{y}$ are the longitudinal acceleration and lateral acceleration, respectively. When the vehicle travels at a constant longitudinal speed, the longitudinal acceleration is close to 0. Therefore, Equation (13) can be further simplified to obtain Equation (14).

(14) $$\left|a_{y}\right|\leq \mu g$$

Under favorable road adhesion conditions, the constraint on acceleration can be relatively relaxed. However, an excessively large constraint range may lead to increased lateral acceleration, adversely affecting occupant comfort. Conversely, overly stringent constraints can result in solver infeasibility during the optimization process. To balance these conflicting requirements, the constraint is formulated as a soft constraint and dynamically adjusted in real time by the solver based on the solution status at each control cycle, as detailed in Equation (15).

(15) $$a_{y,\min}-\varepsilon \leq a_{y}\leq a_{y,\max}+\varepsilon$$

In the equation, $a_{y,\min}$ and $a_{y,\max}$ are the limit of acceleration.

(2) Mass slip angle.

The vehicle’s mass slip angle has a significant impact on driving stability and must be constrained within a reasonable range to ensure safe operation. According to previous studies [36,37], the stable mass slip angle limit can reach ±12° on dry asphalt surfaces with good adhesion, whereas on low-adhesion surfaces such as ice and snow, the limit decreases to approximately ±2°. Exceeding these limits may lead to vehicle rollover or loss of control, highlighting the necessity of enforcing appropriate constraints on the mass slip angle during high-speed trajectory tracking.

(3) Tire slip angle.

In the vehicle dynamics model, the tire slip angle is not treated as a state variable; therefore, it must be calculated at each step of the solution process. According to the tire model equations, the relationship among the mass slip angle, state variables, and control inputs is expressed in Equation (16).

(16) $$\alpha_{f}=\frac{\dot{y}+a\dot{\theta }}{\dot{x}}-\delta_{f}\;\alpha_{r}=\frac{\dot{y}-b\dot{\theta }}{\dot{x}}$$

At any time t, the state variables of the system are known, and the formula for calculating the tire side-slip angle is shown in Equations (17) and (18).

(17) $$\alpha_{f,t}=\frac{{\dot{y}}_{t}+a{\dot{\theta }}_{t}}{{\dot{x}}_{t}}-\delta_{f,t-1}$$

(18) $$\alpha_{r,t}=\frac{{\dot{y}}_{t}-b{\dot{\theta }}_{t}}{{\dot{x}}_{t}}$$

From the tire cornering characteristics, it can be observed that when the tire cornering angle does not exceed 5°, a linear relationship exists between the cornering angle and the cornering force. The development of the vehicle dynamics model is based on the small-angle assumption. Therefore, the constraints on the steering angle of the front wheels must be more stringent, and the specific constraints on them are shown in Equation (19).

(19) $$-\alpha_{\max}<\alpha_{f,t}<\alpha_{\max}$$

This constraint condition is incorporated into the solution process of the quadratic programming problem, but the difference is that this constraint condition does not need to be substituted into the optimization objective function to participate in the optimization calculation.

### 3.2. Design of PID Controller with Forward Feedback

A single control strategy is often insufficient to achieve globally optimal trajectory tracking performance for autonomous vehicles under varying driving conditions. Specifically, MPC may fail to meet tracking requirements in scenarios involving high-speed driving on wet and slippery roads or when computational hardware resources are limited. Therefore, it is necessary to incorporate additional control strategies to complement MPC, ensuring robust and effective trajectory tracking even under non-standard or challenging operating conditions.

PID control is widely applied due to its simplicity, reliable performance, and ease of parameter tuning. Owing to these advantages, PID control is often considered the preferred supplementary control strategy. The PID controller consists of 3 components: proportional, integral, and derivative terms, with each contributing to different aspects of system response and stability. The structure of the PID controller in the joint MPC and PID controller is shown in Figure 1b.

The PID controller first calculates the trajectory tracking error between the reference value and the measured value, as shown in Equation (20).

(20) $$e\left(t\right)=r_{c}\left(t\right)-y\left(t\right)$$

Based on the characteristics of the target control system, the proportional, integral, and derivative components are individually tuned to appropriate values. These three components are then effectively combined to generate the system’s output control signal, which corresponds with the front wheel steering angle of the vehicle.

The PID algorithm expression is shown in Equation (21).

(21) $$u\left(t\right)=K_{P}\cdot e\left(t\right)+K_{I}\int_{0}^{t}e\left(t\right)dt+K_{D}\frac{de\left(t\right)}{dt}$$

where $u\left(t\right)$ is the control quantity, which is the front wheel angle of the vehicle; $K_{p}$ is the proportional part coefficient, and its main function is to quickly eliminate the system deviation; $K_{I}$ is the integral part coefficient, whose main function is to eliminate the system static error; and $K_{D}$ is the differential part coefficient, whose main function is to reflect the system changes. The filtering function is added to the PID controller, so the improved expression of the PID control algorithm is shown in Equation (22).

(22) $$u\left(t\right)=K_{P}\cdot e\left(t\right)+K_{I}\int_{0}^{t}e\left(t\right)dt+K_{D}\frac{de\left(t\right)}{dt}\left(1+K_{F}f\left(t\right)\right)$$

During the controller design process, the PID algorithm first needs to be discretized, as shown in Equation (23).

(23) $$\left\{\begin{array}{l} t=KT\\ \int_{0}^{t}e\left(t\right)dt\approx T\sum_{j=0}^{K}e\left(jT\right)\\ \frac{de\left(t\right)}{dt}\approx \frac{e\left(kT\right)-e\left[\left(k-1\right)T\right]}{T}\\ f\left(t\right)=f\left(kT\right) \end{array}\right.$$

In the formula, the value of T is same as the value of T during discretization in the MPC control algorithm, where $e\left(kT\right)$ will be simplified to $e\left(k\right)$. Substituting Equation (23) into Equation (21), the improved discrete PID control algorithm expression with filtering is obtained, as shown in Equation (24).

(24) $$u\left(k\right)=K_{P}\cdot e\left(k\right)+K_{I}T\sum_{j=0}^{K}e\left(jT\right)+K_{D}\frac{e\left(k\right)-e\left(k-1\right)}{T}\left(1+K_{F}f\left(k\right)\right)$$

The magnitude $e\left(t\right)$ of input deviation of the PID controller is calculated by combining the lateral deviation $y_{e}$ and the heading angle deviation $\theta_{e}$ according to a certain weighted value combination. Because the units of lateral deviation $y_{e}$ and the heading angle deviation $\theta_{e}$ are inconsistent, the whole weighted combination process uses a dimensionless processing method, and the calculation process is shown in Equations (25)–(27).

(25) $${\overset{⌢}{y}}_{e}=1-2\frac{y_{e\max}-y_{e}}{y_{e\max}-y_{e\min}}$$

(26) $${\overset{⌢}{\theta }}_{e}=1-2\frac{\theta_{e\max}-\theta_{e}}{\theta_{e\max}-\theta_{e\min}}$$

(27) $$e\left(t\right)=\rho {\overset{⌢}{y}}_{e}+\left(1-\rho \right){\overset{⌢}{\theta }}_{e}$$

In the formula, ${\overset{⌢}{y}}_{e}$ and ${\overset{⌢}{\theta }}_{e}$ are respectively the lateral deviation and the heading angle deviation after the dimensionless processing; $y_{e\max}$ and $y_{e\max}$ are the maximum and minimum lateral deviations in the track tracking process, respectively; $\theta_{e\max}$ and $\theta_{e\min}$ are the maximum and minimum heading deviations, respectively; $e\left(t\right)$ is the final deviation; and ρ is the deviation weight coefficient. The weight coefficient can be adjusted between 0–1 according to the control requirements.

## 4. Multi-Objective Collaborative Optimization Index

Lateral control is a critical technology for autonomous vehicles. Ensuring vehicle safety is the top priority, followed by improving trajectory tracking accuracy to the greatest extent possible, all while simultaneously maintaining ride comfort and fuel economy. Accordingly, the multi-objective collaborative optimization framework for autonomous vehicles is designed based on four key evaluation criteria: tracking accuracy, comfort, economy, and driving safety.

### 4.1. Tracking Accuracy Evaluation Index

The primary objective of lateral control in autonomous vehicles is to generate a driving trajectory that closely follows the desired path by minimizing a predefined objective function. Accordingly, tracking accuracy can be evaluated based on two key indicators: the fluctuation of tracking error and the accumulated error during the tracking process. The overall trajectory tracking error consists of both lateral displacement deviation and yaw angle deviation. However, due to the difference in units between these two components, normalization of the errors is required prior to evaluation.

The error fluctuation in the tracking process can be described by Equation (28).

(28) $$J_{BDey}=\sqrt{\frac{1}{N}\left(\sum_{k=1}^{N}{\left(\frac{y_{k}\left(t\right)-y_{des}\left(t\right)}{y_{\max}}\right)}^{2}\right)}$$

where $y_{k}\left(t\right)$ is the vehicle lateral position coordinate at time t; $y_{des}\left(t\right)$ is the desired trajectory coordinate at time t; and $y_{\max}$ is the maximum lateral displacement deviation value.

The cumulative value of the tracking process of the lateral error can be described by Equation (29).

(29) $$J_{LJey}=\sqrt{\left(\sum_{N=1}^{k}{\left(\frac{y_{k}\left(t\right)-y_{des}\left(t\right)}{y_{\max}}\right)}^{2}\right)}$$

As before, the fluctuation evaluation index of the yaw angle error is shown in Equation (30).

(30) $$J_{BDe\theta }=\sqrt{\frac{1}{N}\left(\sum_{N=1}^{k}{\left(\frac{\theta_{k}\left(t\right)-\theta_{des}\left(t\right)}{\theta_{\max}}\right)}^{2}\right)}$$

The cumulative value of the tracking process of the yaw angle error can be described by Equation (31).

(31) $$J_{LJe\theta }=\sqrt{\left(\sum_{N=1}^{k}{\left(\frac{\theta_{k}\left(t\right)-\theta_{des}\left(t\right)}{\theta_{\max}}\right)}^{2}\right)}$$

where $\theta_{k}\left(t\right)$ is the actual heading angle of vehicle at time t; $\theta_{des}\left(t\right)$ is the expected heading angle at time t; and $\theta_{\max}$ is the maximum yaw angle deviation value.

The above fluctuation error and cumulative error are synthesized according to the formula, and a comprehensive evaluation index of tracking progress is obtained, as shown in Equation (32).

(32) $$J_{A}=\sqrt{\frac{\omega_{BDe}{J^{2}}_{BDe}+\omega_{HBe}{J^{2}}_{HBe}+\omega_{BD\theta }{J^{2}}_{BDe}+\omega_{HB\theta }{J^{2}}_{HBe}}{\omega_{BDe}+\omega_{HBe}+\omega_{BD\theta }+\omega_{HB\theta }}}$$

In the formula, $\omega_{BDe}$, $\omega_{HBe}$, $\omega_{BD\theta }$, and $\omega_{BD\theta }$ are the error weight coefficients.

### 4.2. Comfort Evaluation Index

In vehicle lateral control, excessive longitudinal acceleration, deceleration, and lateral acceleration can lead to occupant discomfort. In this study, driver and passenger comfort is evaluated based on the maximum longitudinal and lateral accelerations experienced during trajectory tracking, as defined by Equation (33).

(33) $$J_{C}\left(t\right)=\max\sqrt{\frac{\omega_{cax}{\left|\ddot{x}\left(t\right)\right|}^{2}+\omega_{cay}{\left|\ddot{y}\left(t\right)\right|}^{2}}{\omega_{cax}+\omega_{cay}}}$$

In the formula, $\ddot{x}\left(t\right)$ and $\ddot{y}\left(t\right)$ are respectively the maximum longitudinal acceleration and maximum lateral acceleration during the trajectory tracking process, and $\omega_{cax}$ and $\omega_{cay}$ are the weight coefficients.

### 4.3. Vehicle Economy Evaluation Index

Driving economy refers to minimizing the vehicle’s fuel consumption during trajectory tracking. Fuel consumption is closely correlated with the vehicle’s longitudinal acceleration. By reducing longitudinal acceleration, fuel consumption can be effectively decreased during trajectory tracking. In this study, the quadratic terms of longitudinal acceleration and its derivative are employed as indirect evaluation indexes of fuel economy, as shown in Equation (34).

(34) $$J_{E}=\max\sqrt{\rho_{Ea}\cdot a_{lon.}^{2}+\rho_{dEa}\cdot a_{dlon.}^{2}}$$

In the formula, $J_{E}$ is the vehicle economy evaluation index; $\rho_{dEa}$ and $\rho_{Ea}$ are the longitudinal acceleration derivative and the weight coefficient corresponding to the longitudinal acceleration, respectively; and $a_{lon.}$ and $a_{dlon.}$ are the longitudinal acceleration and the longitudinal acceleration derivative, respectively. The vehicle acceleration, the rate of acceleration changes, and frequent changes of engine speed can all be reduced by a minimized $J_{E}$. The fuel economy and fuel consumption during driving can be improved.

### 4.4. Vehicle Safety Indicators

The trajectory tracking control investigated in this study primarily focuses on the lateral control of a single-track vehicle. The safety evaluation indicators are centered on preventing hazardous events such as sliding, rollover, and tail drifting. To address these risks, corresponding constraints are applied to key variables, including the front wheel steering angle, tire–road adhesion conditions, and tire slip angle.

A safety threshold for the vehicle’s lateral acceleration is established to ensure stable operation. The lateral acceleration should remain below or near this threshold under all driving conditions. This safety criterion is evaluated using Equation (35).

(35) $$J_{sa}=\frac{1}{N}\sum_{k=1}^{N}\sqrt{{\left(\frac{{\dot{v}}_{y}\left(k\right)-{\overset{⌢}{\dot{v}}}_{y}}{{\overset{⌢}{\dot{v}}}_{y}}\right)}^{2}}$$

In the formula, ${\dot{v}}_{y}\left(k\right)$ is the lateral acceleration; ${\overset{⌢}{\dot{v}}}_{y}$ is the safety threshold of the longitudinal acceleration; and the value is set for 1 m/s^2^.

In the same way, the side-slip angle of the center of mass is set within a certain safety threshold, and it is evaluated by Equation (36).

(36) $$J_{s\beta }=\frac{1}{N}\sum_{k=1}^{N}\sqrt{{\left(\frac{{\dot{\beta }}_{y}\left(k\right)-{\overset{⌢}{\dot{\beta }}}_{y}}{{\overset{⌢}{\dot{\beta }}}_{y}}\right)}^{2}}$$

where ${\dot{\beta }}_{y}\left(k\right)$ is the vehicle’s center of mass side-slip angle at time k and ${\overset{⌢}{\dot{\beta }}}_{y}$ is the center of mass side-slip angle safety threshold. The setting of this safety threshold is related to the road adhesion coefficient, which is set to 12 when the road adhesion coefficient is about 0.85. When the road adhesion coefficient is small (μ = 0.4), the safety threshold is set to 2°.

Considering the lateral acceleration and the center of mass side-slip angle, the evaluation index of vehicle safety is shown as Equation (37).

(37) $$J_{S}=\sqrt{\frac{\omega_{ca}{J^{2}}_{ca}+\omega_{c\beta }{J^{2}}_{c\beta }}{\omega_{ca}+\omega_{c\beta }}}$$

In Equation (37), $\omega_{ca}$ and $\omega_{c\beta }$ are the error weight coefficients.

## 5. Analysis of Influencing Factors of Control Effect

We validated the performance of the joint MPC and PID controller designed in this study in a Carsim (Version 2023)/MATLAB Simulink (Version R2023b) co-simulation.

**Figure 5:**
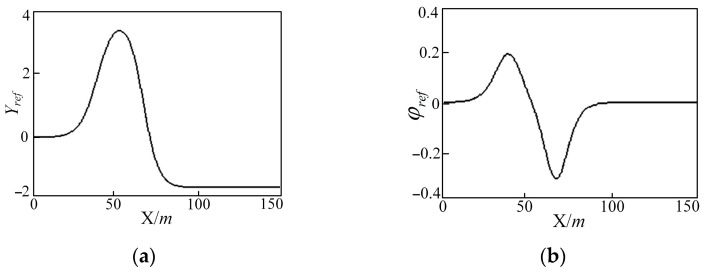
Trajectory diagram and heading angle of the DLC condition: (**a**) trajectory diagram and (**b**) heading angle.

The DLC (Double Lane Change) trajectory and the changes of heading angle are shown in Figure 5. They are composed of reference lateral position $Y_{ref}$ and the reference yaw angle. The independent variables of both the nonlinear functions $Y_{ref}$ and $\varphi_{ref}$ are defined as the lateral position X, as shown in Equations (38) and (39).

(38) $$Y_{ref}\left(X\right)=\frac{d_{y1}}{2}\left(1+\tanh\left(z_{1}\right)\right)-\frac{d_{y2}}{2}\left(1+\tanh\left(z_{2}\right)\right)$$

(39) $$\varphi_{ref}\left(X\right)=\arctan\left(d_{y1}{\left(\frac{1}{\cosh\left(z_{1}\right)}\right)}^{2}\left(\frac{1.2}{d_{x1}}\right)-d_{y2}{\left(\frac{1}{\cosh\left(z_{2}\right)}\right)}^{2}\left(\frac{1.2}{d_{x2}}\right)\right)$$

where $z_{1}=\frac{2.4}{25}\left(X-27.19\right)-1.2$; $z_{2}=\frac{2.4}{21.95}\left(X-56.46\right)-1.2$; $d_{x1}=25$; $d_{x2}=21.95$; $d_{y1}=4.05$; and $d_{y2}=5.7$.

The principle of MPC is to continuously adjust the front wheel steering angle through a control algorithm to minimize the deviation between the actual and reference trajectories, thereby achieving accurate trajectory tracking. However, multiple factors influence the stability of vehicle trajectory tracking. The following three factors are investigated in this study. Meanwhile, the relevant parameters used in the design of the model predictive controller are shown in Table 2.

### 5.1. Influence Law of Road Adhesion Coefficient on Tracking Control

When an autonomous vehicle operates on roads with varying adhesion conditions, its dynamic parameters—such as tire cornering stiffness—change accordingly. These variations significantly affect the performance of the control system. The adhesion coefficient of a normal vehicle on a road surface is generally $\mu \in \left[0.65,0.85\right]$. The adhesion coefficient of a wet and slippery road surface is generally between 0.2 to 0.4. In order to compare and study the influence law of the road adhesion coefficient on the control effect of the controller, the road adhesion condition is divided into three types: dry cement new road, normal road, and wet and slippery road.

The longitudinal speed of the autonomous vehicle is set to 80 km/h. The trajectory tracking controller parameters are set to: sampling time interval T=0.05s, $N_{p}=30$, $N_{c}=20$, $-10{}^{\circ}\leq \delta_{f}\leq 10{}^{\circ}$, and $-1{}^{\circ}\leq \Delta \delta_{f}\leq 1{}^{\circ}$.

First simulation condition: Comparison under varying road adhesion coefficients. The dynamic parameters of an autonomous vehicle differ when operating on roads with different surface conditions. Changes in tire cornering stiffness may result in insufficient lateral force from the ground. Therefore, the vehicle’s trajectory tracking performance under these varying adhesion coefficients must be thoroughly evaluated. Autonomous vehicles were simulated on μ=0.4 (wet and slippery roads), μ=0.65 (normal roads), and μ=0.85 (dry cement new roads).

**Figure 6:**
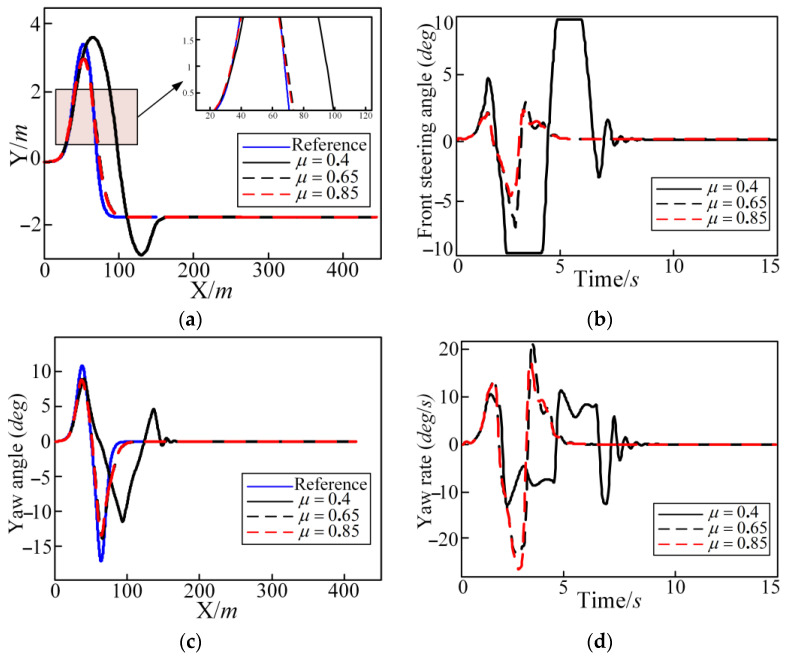
Different adhesion coefficients in the DLC condition regarding the lateral position (Y), front steering angle, yaw angle, and yaw rate: (**a**) lateral position; (**b**) front steering angle; (**c**) yaw angle; and (**d**) yaw rate.

**Figure 7:**
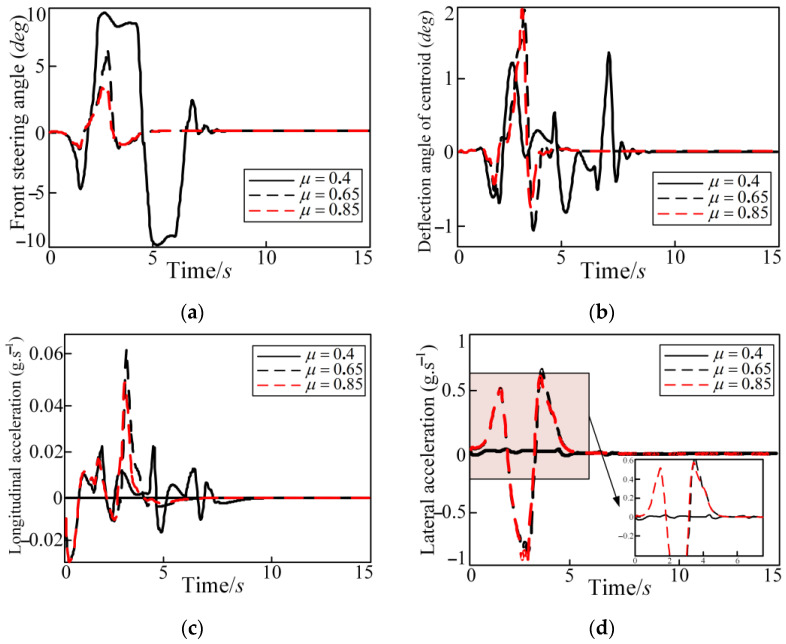
Different adhesion coefficients in the DLC condition regarding the front steering angle, deflection angle of centroid, longitudinal acceleration, and lateral acceleration: (**a**) front steering angle; (**b**) deflection angle of centroid; (**c**) longitudinal acceleration; and (**d**) lateral acceleration.

**Figure 8:**
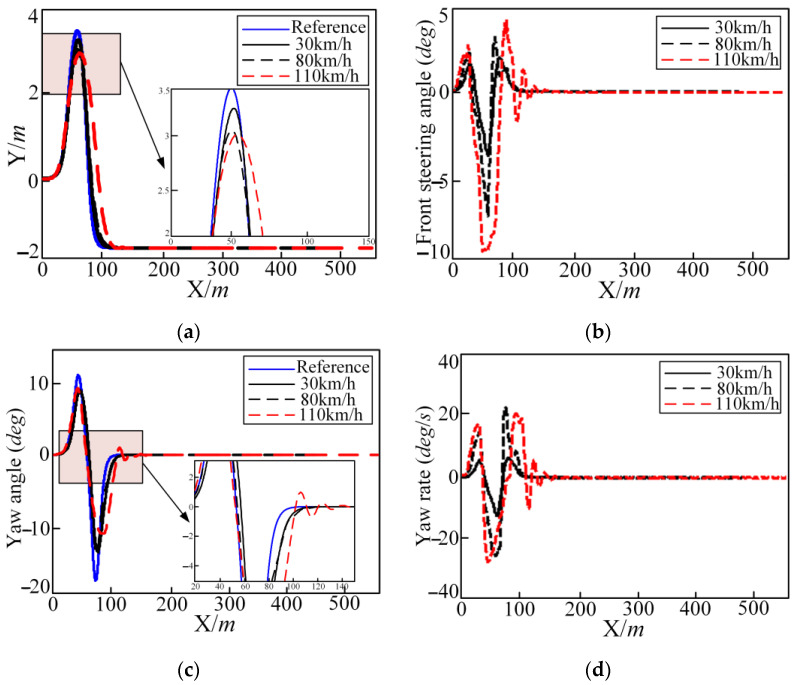
Different longitudinal speeds in the DLC condition regarding the lateral position (Y), front steering angle, yaw angle, and yaw rate: (**a**) lateral position (**b**) front steering angle; (**c**) yaw angle; and (**d**) yaw rate.

The longitudinal speed of vehicle is set to 80 km/h. The reference trajectory is a DLC condition. The simulation results are shown in Figure 6, Figure 7 and Figure 8. Figure 6 shows graphs of the lateral position, front steering angle, yaw angle, and yaw rate with time. Figure 7 shows graphs of the tire slip angle, deflection angle of centroid, longitudinal acceleration, and lateral acceleration with time. In Figure 7 and Figure 8, it can be seen that the autonomous vehicle can basically track the desired trajectory on the roads with different adhesion coefficients. But there is a slight local side slip on the roads with the adhesion coefficient of 0.4. This is because when the vehicle turns on roads with the lower adhesion coefficients, the ground cannot provide the sufficient lateral force. There is a large deviation in the tracking process, as shown in Figure 6 in the lateral position; when μ=0.4, the deviation is near 120 m for the lateral position at 100 m and 150 m. However, Figure 6 and Figure 7 show that the trajectory tracking controller can correct the tracking deviation in time and, finally, converge the tracking deviation to 0. The control quantity and control increment are also within the constraint range to ensure that the actuator can achieve normal implementation and that the driving vehicle has certain comfort and safety requirements. The addition of soft constraints also ensures that the solver can obtain a feasible solution within the specified time. The specific calculation conditions of each evaluation index are shown in Table 3. In Table 3, as the road adhesion coefficient increases, the evaluation indexes of tracking accuracy, economy, and safety become smaller, which indicates that tracking accuracy, economy, and safety are improved. But the index of comfort is worse.

### 5.2. Influence of Driving Speed on Tracking Control of Controller

The driving speed of a vehicle has a certain influence on the control effect of the controller. The model predictive controller is used to predict the future output of the system based on the vehicle model to control the autonomous vehicle. The actual vehicle speed is not constant during the driving process. The vehicle speed varies in a large range. Therefore, a good controller needs to have a good adaptability to control the speed of a vehicle.

The longitudinal speed of the autonomous vehicle is set to 30 km/h, 80 km/h, and 110 km/h, respectively. The road adhesion coefficient is set to μ=0.85. The expected trajectory is set to the DLC condition, and the trajectory tracking controller parameters are set as: sampling time interval T=0.05 s, $N_{p}=30$, $N_{c}=20$, $-10{}^{\circ}\leq \delta_{f}\leq 10{}^{\circ}$, and $-1{}^{\circ}\leq \Delta \delta_{f}\leq 1{}^{\circ}$.

**Figure 9:**
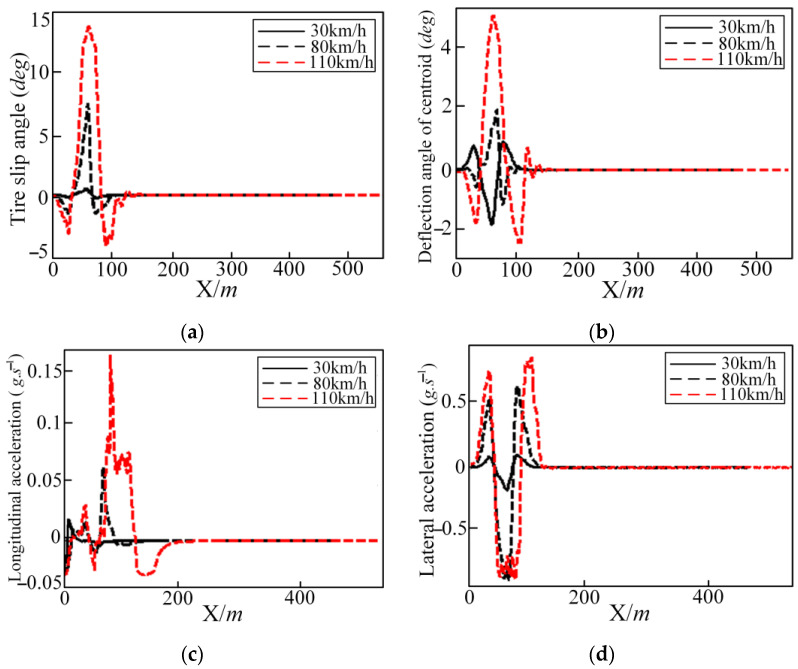
Different longitudinal speeds in the DLC condition regarding the tire slip angle, deflection angle of centroid, longitudinal acceleration, and lateral acceleration: (**a**) tire slip angle; (**b**) deflection angle of centroid; (**c**) longitudinal acceleration; and (**d**) lateral acceleration.

The comparisons of trajectory tracking at three different vehicle speeds are shown in Figure 8 and Figure 9. The control variable lateral position, front steering deflection angle, yaw angle, and yaw rate with time are shown in Figure 8. The change curve of the tire slip angle, deflection angle of centroid, longitudinal acceleration, and lateral acceleration are shown in Figure 8.

It can be seen from Figure 9 that the reference trajectory can be tracked well at different vehicle speeds. But there is a large deviation between the local tracking trajectory and reference trajectory, as shown in Figure 9, at the turning point of 50 m; the yaw angle decreases with the increase in vehicle speed. The front steering angle, yaw rate, tire slip angle, longitudinal acceleration, and centroid deflection angle all increase with the increase in vehicle speed. But the control volume, output volume, and the values of the state quantities are all within the bounds.

In summary, the trajectory tracking controller can basically meet the control requirements within a wide range of vehicle speed changes. Under good adhesion conditions, an increase in the vehicle speed will not cause vehicle instability; but an increase in the vehicle speed will cause the tracking deviation and other evaluation indicators to increase.

The evaluation indicators are shown in Table 4. In Table 4, we can see that under different longitudinal vehicle speeds, the tracking accuracy, comfort, economy, and safety indicators are better at low speeds and that the indicators become worse as the speed increases.

### 5.3. Influence of Tracking Controller Parameters

The selection of controller parameters has a significant impact on the performance of the control system. For MPC algorithms, the prediction horizon and control horizon are among the most critical parameters. To investigate the influence of different prediction and control horizons on trajectory tracking performance, three controllers—designated as A, B, and C—are designed and evaluated accordingly. The parameters of these three controllers are set as follows:

- The parameters of tracking controller A: sampling time interval T=0.05 s, $N_{p}=30$, $N_{c}=20$, $-10{}^{\circ}\leq \delta_{f}\leq 10{}^{\circ}$, and $-1{}^{\circ}\leq \Delta \delta_{f}\leq 1{}^{\circ}$;
- The parameters of tracking controller B: sampling time interval T=0.05 s, $N_{p}=25$, $N_{c}=10$, $-10{}^{\circ}\leq \delta_{f}\leq 10{}^{\circ}$, and $-1{}^{\circ}\leq \Delta \delta_{f}\leq 1{}^{\circ}$;
- The parameters of tracking controller C: sampling time interval T=0.05 s, $N_{p}=15$, $N_{c}=10$, $-10{}^{\circ}\leq \delta_{f}\leq 10{}^{\circ}$, and $-1{}^{\circ}\leq \Delta \delta_{f}\leq 1{}^{\circ}$.

The control parameters of three controllers A, B, and C are that the prediction time domain and the control time domain are successively smaller. However, the other parameters are kept the same; the road adhesion coefficient is μ=0.85; and the longitudinal vehicle speed is set to 80 km/h. Under the action of different controllers, the trajectory tracking and stable driving ability of the autonomous vehicle is verified.

**Figure 10:**
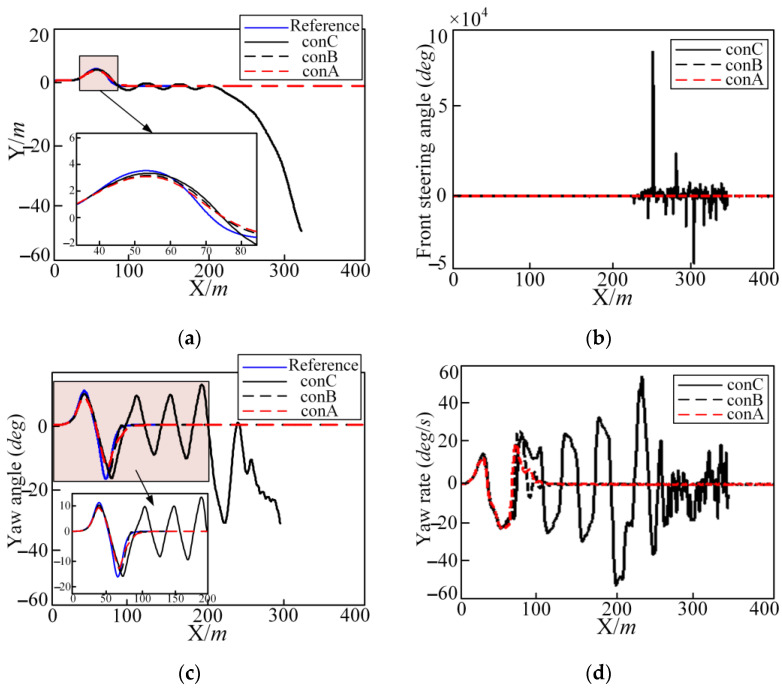
Different controller parameters in the DLC condition regarding the lateral position (Y), front steering angle, yaw angle, and yaw rate: (**a**) the lateral position; (**b**) front steering angle; (**c**) yaw angle; and (**d**) yaw rate.

The simulation results under different controller parameters in the DLC condition are shown in Figure 10. The comparisons of trajectory tracking results for different controllers are shown in Figure 10, which also includes the trajectory tracking process, lateral position, front steering angle, yaw angle, and yaw rate. It can be seen from Figure 10 that both controllers A and B can basically track the desired trajectory and eventually converge the tracking deviation to 0, while the control effect of controller C is basically out of control.

When comparing controllers A and B—although the parameters of these two controllers can better track the desired trajectory—controller A has a smaller tracking deviation than the controller B. However, the advantage of controller B is that the calculation time is much less.

In summary, the lengths of the prediction horizon and control horizon significantly influence the tracking accuracy of the trajectory tracking controller. However, they also have a substantial impact on the real-time computational burden. A longer control horizon increases the computational time, which may compromise the real-time performance required by autonomous vehicles. Therefore, a larger prediction or control horizon does not necessarily lead to improved control performance. The selection of these parameters must strike a balance between tracking accuracy, available computational resources, and real-time operational constraints.

The specific calculation settings for each evaluation index are presented in Table 5. As shown in this table, for controllers A, B, and C, the indicators related to tracking accuracy, comfort, economy, and safety progressively deteriorate as the prediction horizon and control horizon are reduced. Therefore, simulation results reveal a general trend: under the premise that real-time performance and computational requirements are satisfied, increasing the prediction and control horizons leads to improved overall control performance.

### 5.4. Simulation Results of Combined Controller of MPC and PID Control on Wet and Slippery Roads

**Figure 11:**
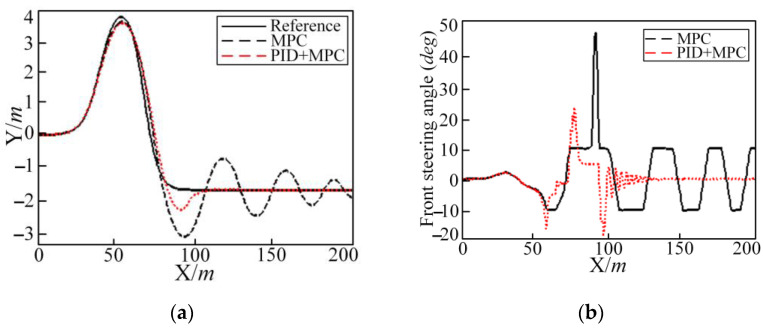

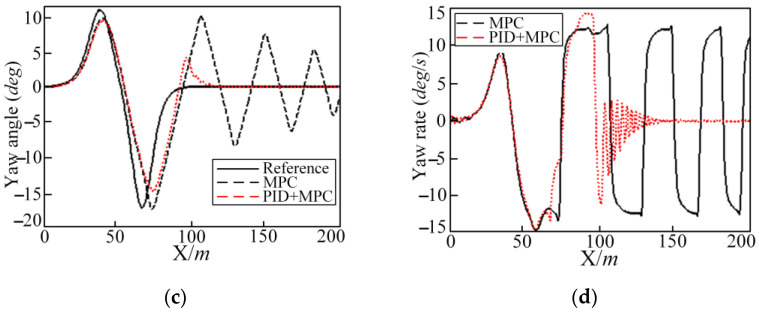
Different control methods in the DLC condition regarding the lateral position (Y), front steering angle, yaw angle, and yaw rate: (**a**) the lateral position; (**b**) front steering angle; (**c**) yaw angle; and (**d**) yaw rate.

**Figure 12:**
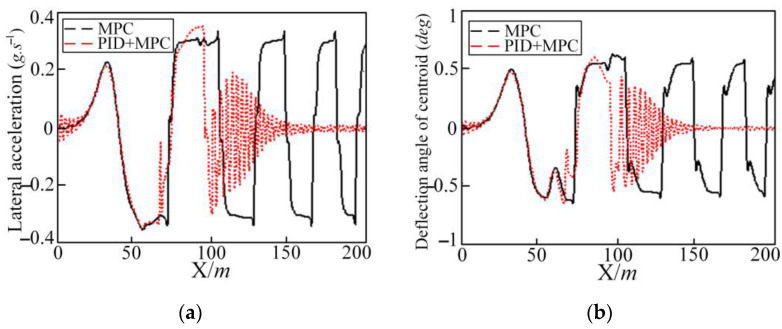
Different control methods in the DLC condition regarding the lateral acceleration and deflection angle of centroid: (**a**) lateral acceleration and (**b**) deflection angle of centroid.

Through the simulation results under different road adhesion coefficients, driving speeds, and controller parameters, it can be seen that on wet and slippery roads (road adhesion coefficient is 0.4) and that where the controller hardware conditions are not high and the vehicle driving speed is high at 45 km/h, the trajectory tracking vehicle under the model predictive controller will exhibit shimmy. In Figure 11, the vehicle longitudinal simulation speed is set to 50 km/h. Under the pure MPC method, the shimmy phenomenon should begin to appear near the coordinate of 80 m. The front steering angle, the vehicle yaw angle, and the yaw rate are shown in Figure 11. The lateral acceleration and deflection angle of centroid slip in Figure 12 both have the same changing law.

By using the PID and MPC joint controller for simulation, the MPC controller parameters are set to $N_{p}=30$ and $N_{c}=20$, the longitudinal vehicle speed is set to 50 km/h, and the road adhesion coefficient μ=0.4. The effects of the PID and MPC joint controller are compared.

In Figure 11, a graph of the lateral position is presented after the PID joint controller is added. The shimmy phenomenon of each simulation quantity is obviously optimized. The whole simulation quantity converges over time. In Table 6, the tracking accuracy index has increased by 36.3%. The comfort, economy, and safety indexes have increased by 38.6%, 19.8%, and 8.7%, respectively. The specific calculation of each evaluation index is shown in Table 6. In Table 6, we can see that each evaluation index with PID control is better than without PID control.

According to the parameter influence rule obtained by the previous simulation, in order to obtain a better control effect, we changed the model predictive controller parameters to $N_{p}=25$ and $N_{c}=10$, the road adhesion coefficient to μ=0.4, and the longitudinal vehicle speed to 50 km/h.

**Figure 13:**
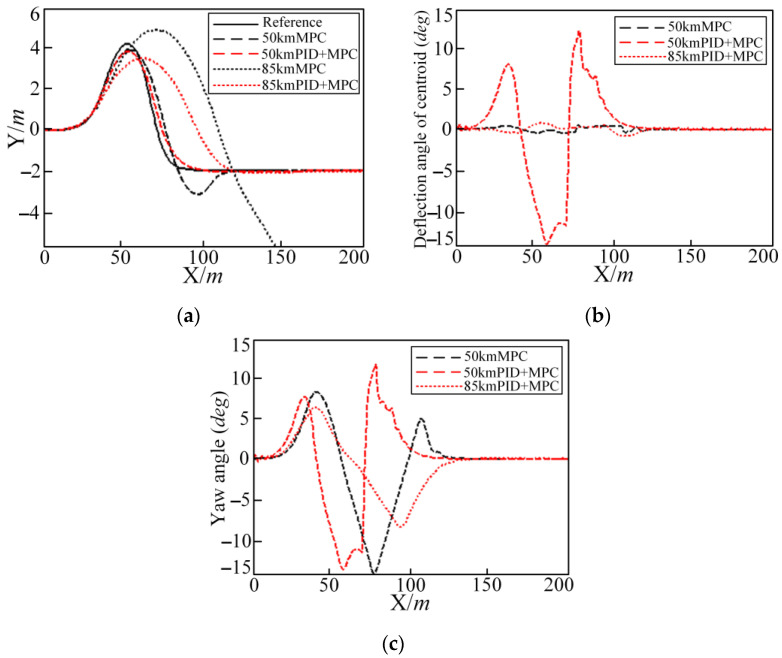
Simulation results for MPC and PID + MPC controller in DLC conditions at 50 km/h and 85 km/h: (**a**) lateral position; (**b**) deflection angle of centroid; and (**c**) yaw angle.

Trajectory tracking under the model prediction control shows a slight shimmy phenomenon near the longitudinal coordinate of 80 m. When the vehicle speed is 85 km/h, as shown in Figure 13, the vehicle has already lost control. In order to improve the control effect, the PID and MPC joint controller method is used.

The longitudinal simulation speed is 50 km/h and 85 km/h, respectively, by using the PID and MPC joint controller for simulation, comparing with or without the PID joint controller effect, the simulation results are shown in Figure 13.

In Figure 13, after the PID joint controller is added, the shimmy phenomenon of each simulation quantity at 50 km/h is optimized. The vehicle out-of-control phenomenon disappears at 85 km/h. Although the center of mass slip angle and the vehicle yaw angle are both large, they are within the vehicle safety limit. In Table 7 and Table 8, after adding the PID joint controller, the tracking accuracy index at 50 km/h is increased by 53.9%, and the comfort, economy, and safety indexes are increased by 87.4%, 52.1%, and 47.5%, respectively. For 85 km/h, the tracking accuracy index is increased by 22.2%, and the comfort, economy, and safety indexes are increased by 9.4%, 51%, and 5.3%, respectively. The specific calculation of each evaluation index is shown in Table 7 and Table 8.

**Figure 14:**
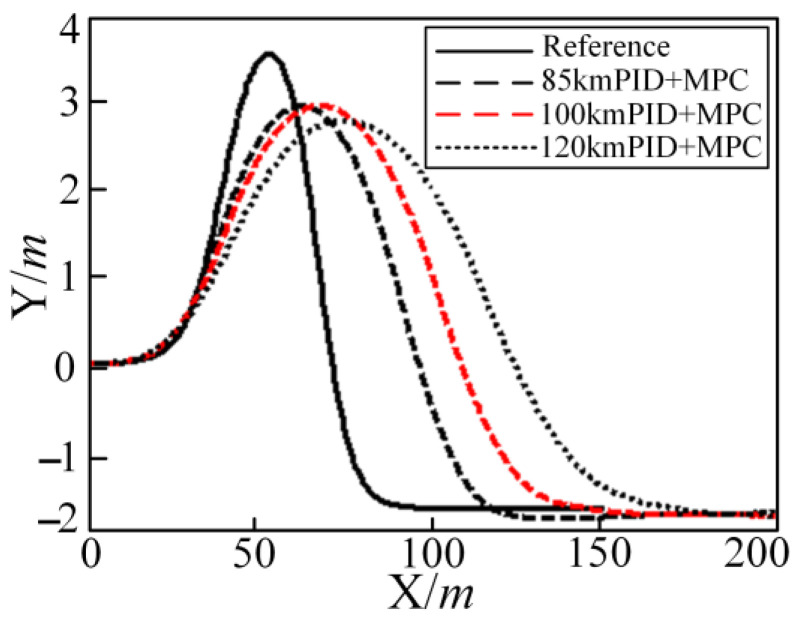
Simulation results for reference and PID + MPC controller in the DLC condition at 85 km/h, 100 km/h, and 120 km/h.

In order to further illustrate the advantages of the combined controller method of PID control and MPC, the control effect is simulated under the driving speed of 100 km/h and 120 km/h. The combined control effects of 85 km/h, 100 km/h, and 120 km/h are shown in Figure 14. It can be seen from Figure 14 that the control effect of high-speed driving on the low-adhesion roads is obviously optimized and controlled.

## 6. Conclusions

Through the design of a trajectory tracking controller under high-speed driving conditions, the influences of the road adhesion coefficient, longitudinal vehicle speed, and controller parameters on the control effect of the controller are analyzed. The PID and MPC joint controller methods are proposed, and the work of this chapter has the following three main aspects.

1. The vehicle dynamics model is used as the basis of the predictive model controller. The trajectory tracking controller under the high-speed driving conditions is designed to improve the controller’s ability to predict the future behavior of the vehicle and ensure the stability of the vehicle. In order to achieve a good multi-objective cooperative control effect, the control constraints, state constraints, and the initial and end state constraints are imposed on the control objective function. The multi-objective performance evaluation indicators are designed.
2. The simulation test is carried out on the DLC condition. The influence of the road adhesion coefficient, driving speed, and controller parameters on the control effect under different driving conditions are analyzed. The evaluation index values are calculated to compare and analyze the influence of the various factors on the control effect of the controller.
3. The combined controller method of PID control and MPC is proposed when the level of wet and slippery roads and the controller hardware is low. However, the research results show that the simple MPC controller has a better control effect on good roads and that the combined controller of PID control and MPC can make up for shortcomings of simple the MPC controller under high-speed driving conditions on wet and slippery roads, making the vehicle trajectory tracking more efficient, comfortable, and economical. The safety control indicators have been significantly optimized. The controller parameters are set to $N_{p}=15$ and $N_{c}=10$; the longitudinal vehicle speed is 50 km/h; and the road adhesion coefficient μ=0.4. After adding the PID joint controller, the tracking accuracy index is increased by 36.3%. An examination of the data reveals a marked increase in comfort, economy, and safety indicators, with respective growth rates of 38.6%, 19.8%, and 8.7%. The controller parameters are $N_{p}=25$, $N_{c}=10$, the road adhesion coefficient μ=0.4, and the tracking accuracy index is increased by 53.9% at 50 km/h. An examination of the data reveals a marked increase in comfort, economy, and safety indicators, with respective growth rates of 87.4%, 52.1%, and 47.5%. At 85 km/h, the tracking accuracy index is increased by 22.2%. An examination of the data reveals a marked increase in comfort, economy, and safety indicators, with respective growth rates of 9.4%, 51%, and 5.3%. Based on the above experimental data, it is shown that the joint controller of MPC and PID control can effectively improve the comprehensive performance of the path tracking control of autonomous vehicles when driving at high speeds on wet and slippery roads. This work provides an effective engineering practice reference for the research of autonomous vehicles under complex working conditions.

## Figures and Tables

**Table 1:** Technical comparison table. In Control Accuracy, “↑” stands for increased accuracy and “↓” stands for decreased accuracy; in Computation Time, “↑” stands for longer computation time and “↓” stands for shorter computation time, where “→” indicates that the accuracy or computation time remains constant.

Technical Approach	Key Parameters	Control Accuracy	Computation Time
Optimal model	Dimensions of the vehicle model Linearization assumption	↓	↓
predictive time domain adjustment	N (Predictive time domain)	↑ (N↑) ↓ (N↓)	↑ (N↑) ↓ (N↓)
Control period adjustment	Control period	↑ (High Frequency PID) ↓ (Low frequency MPC)	↑ (High Frequency PID) ↓ (Low frequency MPC)
Hybrid control structure	Controller division of labor and interconnection mechanisms	↑ →	↓ →

**Table 2:** MPC controller parameters.

Parameter/Unit	Value
Simulation step/s	0.05
Road adhesion coefficient	0.85/0.65/0.4
Weight coefficient *Q*	$\left[\begin{array}{ccc} 200 & 0 & 0\\ 0 & 100 & 0\\ 0 & 0 & 100 \end{array}\right]$
Control increment weight coefficient *R*	1.1 × 10^5^
Control factor weighting coefficient *ρ*	1000
$\delta_{f}$	$\left[-10{}^{\circ},10{}^{\circ}\right]$
$\Delta \delta_{f}$	$\left[-1{}^{\circ},1{}^{\circ}\right]$

**Table 3:** Comparison of evaluation indexes for control of DLC conditions with different pavement adhesion coefficients.

Evaluation Index		μ=0.4	μ=0.65	μ=0.85
Tracking Accuracy	$J_{BDey}$	0.4464	0.0864	0.0789
	$J_{LJey}$	7.9986	1.5482	1.4138
	$J_{BDe\theta }$	0.6510	0.1609	0.1509
	$J_{LJe\theta }$	11.6641	2.8829	2.7036
	$J_{Final}$	7.0826	1.6387	1.5279
Comfort	$J_{c}$	0.0299	0.6066	0.6926
Economy	$J_{e}$	0.0212	0.0472	0.0365
Safety	$J_{s\beta }$	1.0178	0.9542	0.9586
	$J_{sa}$	1.0042	1.2001	1.0421
	$J_{s}$	1.0110	1.0841	1.0012

**Table 4:** Comparison of control evaluation indexes for DLC conditions of different longitudinal speeds.

Evaluation Index		30 km/h	80 km/h	110 km/h
Tracking Accuracy	$J_{BDey}$	0.0688	0.0864	0.2802
	$J_{LJey}$	1.9473	1.5482	4.1464
	$J_{BDe\theta }$	0.2568	0.1609	0.3431
	$J_{LJe\theta }$	7.2692	2.8829	5.0780
	$J_{Final}$	1.6387	3.2854	3.7651
Comfort	$J_{c}$	0.1236	0.6066	0.6117
Economy	$J_{e}$	0.0215	0.0472	0.1216
Safety	$J_{s\beta }$	0.9996	0.9542	1.1400
	$J_{sa}$	1.0000	1.2001	1.4010
	$J_{s}$	0.9998	1.0841	1.2772

**Table 5:** Comparison of control evaluation indexes of different controller parameters in DLC conditions.

Evaluation Index		Controller A	Controller B	Controller C
Tracking Accuracy	$J_{BDey}$	0.0864	0.0814	0.1655
	$J_{LJey}$	1.5482	1.4580	2.9648
	$J_{BDe\theta }$	0.1609	0.1833	0.5293
	$J_{LJe\theta }$	2.8829	3.2842	9.4838
	$J_{Final}$	1.6387	1.7994	4.9759
Comfort	$J_{c}$	0.6066	0.6152	0.6551
Economy	$J_{e}$	0.0472	0.0500	0.0765
Safety	$J_{s\beta }$	0.9542	1.0109	0.9841
	$J_{sa}$	1.2001	1.2244	1.5305
	$J_{s}$	1.0841	1.1227	1.2866

**Table 6:** MPC and PID + MPC controller evaluation index comparisons.

Evaluation Index		MPC	PID + MPC
Tracking Accuracy	$J_{BDey}$	0.1701	0.0812
	$J_{LJey}$	3.7220	1.7775
	$J_{BDe\theta }$	0.4767	0.3125
	$J_{LJe\theta }$	10.4335	6.8390
	$J_{Final}$	5.5445	3.536
Comfort	$J_{c}$	0.2494	0.1531
Economy	$J_{e}$	0.0545	0.0437
Safety	$J_{s\beta }$	1.0002	0.9991
	$J_{sa}$	0.9705	0.8667
	$J_{s}$	0.9855	0.8996

**Table 7:** MPC and PID + MPC controller evaluation index comparison (50 km/h).

Evaluation Index		MPC	PID + MPC
Tracking Accuracy	$J_{BDey}$	0.0794	0.1154
	$J_{LJey}$	1.7374	2.5254
	$J_{BDe\theta }$	0.5090	0.2076
	$J_{LJe\theta }$	11.1398	4.5444
	$J_{Final}$	5.6431	2.6022
Comfort	$J_{c}$	0.2433	0.0306
Economy	$J_{e}$	0.0453	0.0217
Safety	$J_{s\beta }$	2.5105	1.0062
	$J_{sa}$	1.0006	1.0018
	$J_{s}$	1.9110	1.0040

**Table 8:** MPC and PID + MPC controller evaluation index comparison (85 km/h).

Evaluation Index		MPC	PID + MPC
Tracking Accuracy	$J_{BDey}$	0.6760	0.4350
	$J_{LJey}$	11.3715	7.3175
	$J_{BDe\theta }$	0.8610	0.7327
	$J_{LJe\theta }$	14.4834	12.3253
	$J_{Final}$	9.2233	7.1796
Comfort	$J_{c}$	0.2917	0.2642
Economy	$J_{e}$	0.2062	0.1011
Safety	$J_{s\beta }$	1.0039	0.9976
	$J_{sa}$	1.1030	1.0002
	$J_{s}$	1.0546	0.9989

## Data Availability

Data are contained within the article.
